# Peripheral Inflammation Featuring Eosinophilia or Neutrophilia Is Associated with the Survival and Infiltration of Eosinophils within the Tumor among Various Histological Subgroups of Patients with NSCLC

**DOI:** 10.3390/ijms25179552

**Published:** 2024-09-03

**Authors:** Bilal Alashkar Alhamwe, Kadriya Yuskaeva, Friederike Wulf, Frederik Trinkmann, Mark Kriegsmann, Michael Thomas, Corinna Ulrike Keber, Elke Pogge von Strandmann, Felix J. Herth, Saeed Kolahian, Harald Renz, Thomas Muley

**Affiliations:** 1 Institute of Laboratory Medicine, German Center for Lung Research (DZL), Universities of Giessen and Marburg Lung Center (UGMLC), Medical Faculty, Philipps University of Marburg, 35043 Marburg, Germany; friederike.wulf@e-mail.de (F.W.); saeed.kolahian@uni-marburg.de (S.K.); harald.renz@uk-gm.de (H.R.); 2 Institute of Tumor Immunology, Center for Tumor Biology and Immunology, Philipps University Marburg, 35043 Marburg, Germany; poggevon@staff.uni-marburg.de; 3 College of Pharmacy, International University for Science and Technology (IUST), Daraa 15, Syria; 4 Translational Lung Research Center (TLRC), German Center for Lung Research (DZL), 35394 Heidelberg, Germany; kadriya.yuskaeva@med.uni-heidelberg.de (K.Y.); frederik.trinkmann@med.uni-heidelberg.de (F.T.); kriegsmann@pathologie-wiesbaden.de (M.K.); michael.thomas@med.uni-heidelberg.de (M.T.); felix.herth@med.uni-heidelberg.de (F.J.H.); 5 Translational Research Unit (STF), Thoraxklinik, University Hospital Heidelberg, 69126 Heidelberg, Germany; 6 Department of Pneumology and Respiratory Medicine, Thoraxklinik, University Hospital Heidelberg, 69126 Heidelberg, Germany; 7 Department of Biomedical Informatics (DBMI), Center for Preventive Medicine and Digital Health Baden-Württemberg (CPD-BW), University Medical Center Mannheim, Medical Faculty Mannheim, Heidelberg University, 69117 Heidelberg, Germany; 8 Institute of Pathology, University Hospital Heidelberg, Pathology Wiesbaden, Ludwig-Erhard-Str. 100, 65199 Wiesbaden, Germany; 9 Department of Oncology, Thoraxklinik, University Hospital Heidelberg, 69126 Heidelberg, Germany; 10 Institute for Pathology, University Hospital Giessen and Marburg, 35037 Marburg, Germany

**Keywords:** lung adenocarcinoma, lung non-adenocarcinoma, eosinophilia and neutrophilia, eosinophils–tumor infiltration, prognosis

## Abstract

Immune activation status determines non-small cell lung cancer (NSCLC) prognosis, with reported positive/negative associations for T helper type 2 (TH2) responses, including allergen-specific IgE and eosinophils. Our study seeks to explore the potential impact of these comorbid immune responses on the survival rates of patients with NSCLC. Our retrospective study used data from the Data Warehouse of the German Center for Lung Research (DZL) and Lung Biobank at Thoraxklinik Heidelberg. We estimated the association of blood eosinophilia and neutrophilia on survival rates in an inflammatory cohort of 3143 patients with NSCLC. We also tested sensitization to food and inhalants and high-sensitivity C-reactive protein (hs-CRP) in a comorbidity cohort of 212 patients with NSCLC. Finally, we estimated the infiltration of immune-relevant cells including eosinophils, T-cells, and mast cells in a tissue inflammatory sub-cohort of 60 patients with NSCLC. Sensitization to at least one food or inhalant (sIgE) was higher in patients with adenocarcinoma (adeno-LC) than the non-adenocarcinoma (non-adeno-LC). Furthermore, hs-CRP was higher in non-adeno-LC compared with adeno-LC. Peripheral inflammation, particularly eosinophilia and neutrophilia, was associated with poor survival outcomes in NSCLC with a clear difference between histological subgroups. Finally, blood eosinophilia was paralleled by significant eosinophil infiltration into the peritumoral tissue in the lung. This study provides novel perspectives on the crucial role of peripheral inflammation, featuring eosinophilia and neutrophilia, with overall survival, underscoring distinctions between NSCLC subgroups (adeno-LC vs. non-adeno-LC). Peripheral eosinophilia enhances eosinophil infiltration into tumors. This sheds light on the complex interplay between inflammation, eosinophil infiltration, and NSCLC prognosis among various histological subtypes. Further studies are required to underscore the role of eosinophils in NSCLC among different histological subgroups and their role in shaping the tumor microenvironment.

## 1. Introduction

Lung cancer is a leading cause of cancer-related deaths worldwide with a five-year survival rate of 17.8% [1]. In 2018, the German Centre for Cancer Registry Data (ZfKD) recorded approximately 57,000 new diagnoses of lung cancer in Germany [2] and the number of cases is predicted to increase by 2022 [2]. Histologically, NSCLC accounts for approximately 85% of all lung cancer cases, with two predominant subtypes (adenocarcinoma and squamous cell carcinoma); small cell lung cancer (SCLC) accounts for the other 15% [3,4,5]. 

Comorbidities, such as bronchial asthma and chronic obstructive pulmonary disease (COPD), significantly impact lung cancer survival rates [6,7] and are being utilized in new approaches to risk stratification [8,9]. Allergo-oncology studies explore associations between lung cancer and allergies [10,11,12,13]. Potential links have been postulated between elevated levels of allergen-specific immunoglobulin E (sIgE) and increased risk of certain cancers, such as non-Hodgkin lymphoma, esophageal, oropharyngeal, and lung [14]. Studies have also found that patients with malignancies exhibit elevated levels of blood markers of inflammation, such as hs-CRP, suggesting potential as diagnostic markers for cancer, particularly NSCLC [15,16,17]. However, the exact prevalence of these peripheral inflammatory markers in adeno-LC and non-adeno-LC is still uncertain. Increasing blood levels of sIgE and hs-CRP can result in peripheral inflammation, characterized by elevated levels of eosinophils or neutrophils in the blood, respectively [18,19,20]. This suggests a persistent immune response, which may impact the prognosis in both adeno-LC and non-adeno-LC.

Thus, we hypothesized that peripheral blood inflammation with eosinophils or neutrophils is associated with the overall survival of patients with lung cancer in different histological subgroups including adeno-LC and non-adeno-LC. Peripheral blood inflammation may trigger a stromal response, reflected by changes in the infiltration of inflammatory immune cells, most importantly eosinophils, T-cells, and mast cells, into the peritumor and intratumor regions of lung cancer. This is considered to influence the biological behavior of the tumor. 

## 2. Results

**Figure 1:**
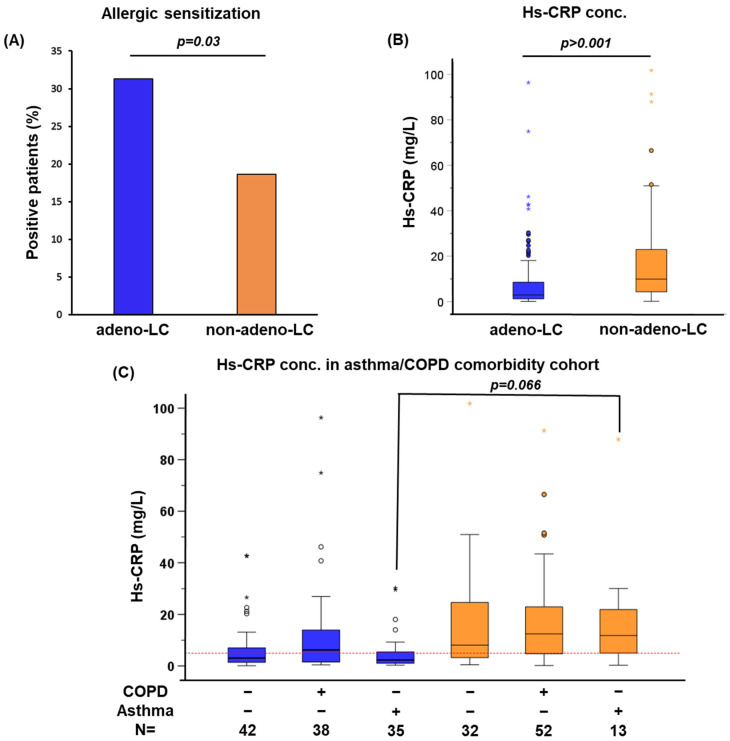
**Chronic inflammatory biomarkers in adeno-LC vs. non-adeno-LC**. (**A**) Patients sensitized to at least one inhalant or food allergen (sIgE > 0.35 kU/L). Specific IgE was measured for a representative panel of inhalant allergens (n = 8) and food allergens (n = 6) in the sera of NSCLC patients (n = 212), of which n = 115 had adeno-LC and n = 97 non-adeno-LC. Significant differences were tested using the Mann–Whitney U-test. (**B**) Distribution of hs-CRP concentrations in adeno-LC and non-adeno-LC. Significant differences were tested using the Mann–Whitney U-test. (**C**) Distribution of hs-CRP concentrations considering the histology (adeno-LC vs. non-adeno-LC) and lung comorbidities (asthma, COPD, and neither). Both stars and cycles describe outliers. Among these outliers, stars specifically mark extreme values. The numbers of patients in each group are shown. Significant differences were tested using the Kruskal–Wallis test.

The presence of allergen-specific IgE in plasma is an important biomarker for atopic sensitization. Although this sub-cohort has a limited number of asthmatic and COPD cases, we used the asthma/COPD comorbidity cohort to assess the association between atopic sensitization and NSCLC histological subgroups. Plasma sIgE of patients with NSCLC was measured against a representative panel of inhalant and food allergens, and individuals with sIgE > 0.35 kU/L were considered sensitized. A significantly higher proportion of patients with adeno-LC were sensitized compared with non-adeno-LC (*p* = 0.03; Figure 1A). This implies a potentially important role of sIgE in adeno patients.

We further measured hs-CRP, a blood biomarker used as an indicator for subclinical inflammation and acute phase response, we measured this in the blood of the same cohort. Our results indicated a significant increase in hs-CRP in non-adeno-LC compared with adeno-LC (*p* > 0.001; Figure 1B), indicating subclinical inflammation in the patients with non-adeno-LC.

Next, we studied the link between asthma or COPD comorbidity and blood hs-CRP in patients with NSCLC, specifically within the histological subgroups of adeno-LC and non-adeno-LC. A global increase in the blood level of hs-CRP was seen in non-adeno-LC compared with adeno-LC, regardless of comorbidities (Figure 1C). These data suggest the presence of systemic inflammation in patients with non-adeno-LC regardless of asthma and COPD comorbidities. 

To analyze the association between eosinophilia and neutrophilia and NSCLC survival, we assessed 3143 patients with NSCLC across the two histological subgroups (adeno-LC vs. non-adeno-LC). The histological groups differed significantly by gender, age, body mass index (BMI), cancer stage, smoking status, and the presence of blood eosinophilia/neutrophilia (*p*-values were calculated using chi-squared and median tests and are listed in Table 1). More details on patient criteria and demographic data are provided in Materials and Methods and Table 1. 

**Figure 2:**
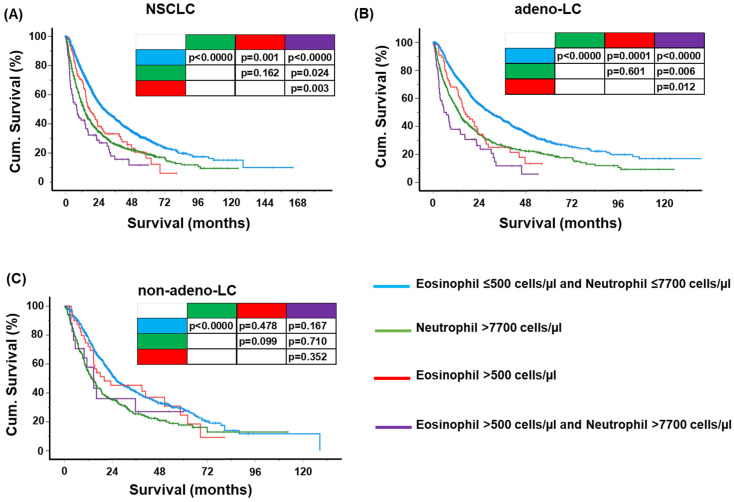
**Impact of peripheral inflammation on cumulative survival.** (**A**–**C**) Cumulative survival in patients with NSCLC with respect to peripheral blood eosinophil and neutrophil counts. Blue line: blood eosinophil ≤ 500 cells/µL and blood neutrophil ≤ 7700 cells/µL. Green line: neutrophilia (blood neutrophil > 7700 cells/µL). Red line: eosinophilia (blood eosinophil > 500 cells/µL). Purple line: both eosinophilia and neutrophilia (blood eosinophil > 500 cells/µL and blood neutrophil > 7700 cells/µL). Cumulative survival was tested for all patients with NSCLC (**A**); those with adeno-LC (**B**); and those with non-adeno-LC (**C**). Univariate results of pairwise comparison (log-rank test) were listed in the figure. For results of multivariate regression analyses adjusting for age, gender, stage, BMI, and smoking status, refer to Table 2 and Table 3.

We analyzed the lung cancer cohort for eosinophilia alone, neutrophilia alone, or both eosinophilia plus neutrophilia using cut-off values of 500 eosinophils and 7700 neutrophils per microliter of blood. The association between pre-therapeutically increased eosinophils, neutrophils, or both and overall survival is shown in Figure 2A–C, Table 2 and Table 3. 

All patient groups with blood eosinophilia or neutrophilia had significantly lower survival rates compared with patients with normal leukocyte counts. Patients with combined neutrophilia and eosinophilia showed the worst outcome (Figure 2A). Multivariate analyses controlling for gender, age, BMI, histology, stage, and smoking status confirmed eosinophilia and/or neutrophilia as a significant independent prognostic factor in NSCLC (Table 2). 

**Figure 3:**
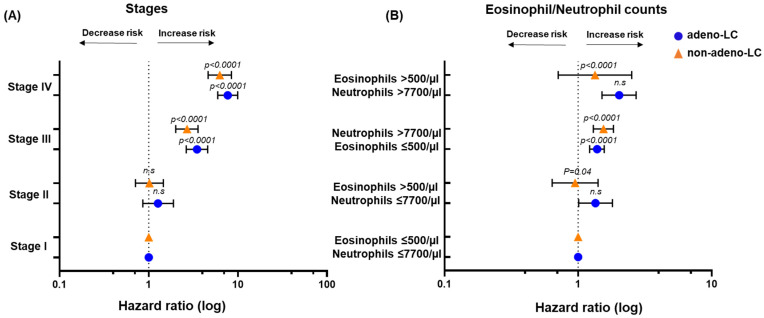
**Hazard ratios using Cox regression analysis model.** (**A**,**B**) The hazard ratio and overall survival were calculated according to subgroups (adeno-LC and vs. non-adeno-LC) controlling for tumor stage (**A**) and blood eosinophilia and neutrophilia (**B**). Hazard ratios were derived from a Cox regression analysis model.

Remarkably, there was a clear difference between patients with adeno-LC and non-adeno-LC (Figure 2B,C; Table 3). Although eosinophilia (eosinophils > 500/µL and neutrophils ≤ 7700/µL; HR = 1.35), neutrophilia (neutrophils > 7700/µL and eosinophils ≤ 500/µL; HR = 1.39), and combined eosinophilia and neutrophilia (eosinophils > 500/µL and neutrophils > 7700/µL; HR = 2.03) were found to be significant prognostic factors in the advanced stages of adeno-LC (Figure 3A,B) and (Table 2), only neutrophilia (HR = 1.55) was significant in the non-adeno-LC subgroup in the advanced stages (Figure 3A,B) and Table 2. The hazard ratio (HR), 95% confidence interval, and *p*-values for all comparisons are provided in Table 2. Selected survival characteristics (median survival, 5-year survival rate) for the whole cohort and the histological subgroups depicted in Figure 2A–C are summarized in Table 3. The multivariate *p*-values are given for the comparison between each subgroup and the group of patients with normal leukocyte values, respectively. Although eosinophilia or neutrophilia was a highly significant prognostic factor in our retrospective lung cancer cohort study, surprisingly it had only marginal use for survival prediction in new patients using time-dependent prediction error (Brier score; Figure S1A,B) and time-dependent ROC analysis (Figure S1C,D). The major discriminating effect of eosinophil or neutrophil counts was seen within the first 20 months after diagnosis (Figure S1C,D).

The infiltration of immune cells in the tumor microenvironment (TME) might be influenced by peripheral inflammation featuring eosinophilia or neutrophilia. Therefore, we assessed the infiltration of eosinophils, mast cells, and T cells into the peritumoral and intratumoral regions of NSCLC tumor tissues from resected patients in the “eosinophilia sub-cohort”. More details on the cohort are provided in the Section 4. 

**Figure 4:**
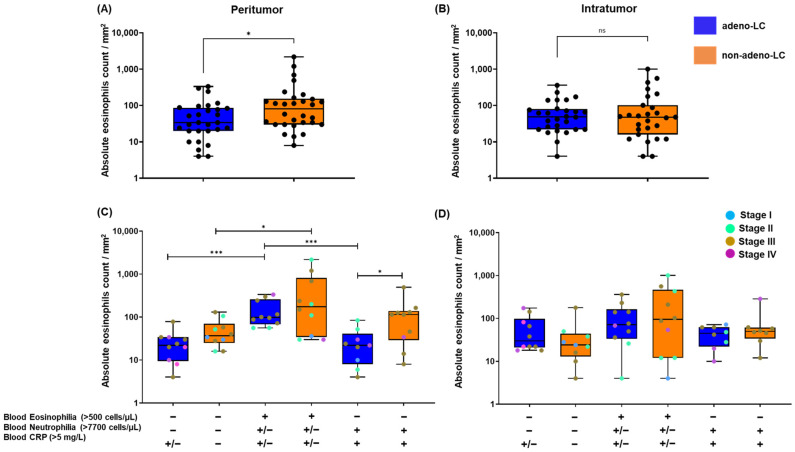
**Distribution of eosinophils in lung cancer tissue**. (**A**,**B**) The distribution of infiltrated eosinophils in adeno-LC vs. non-adeno-LC, regardless of eosinophilia or neutrophilia status, in peritumoral (**A**) and intratumoral (**B**) regions. (**C**,**D**) The infiltration of eosinophils between adeno-LC and non-adeno-LC with respect to peripheral eosinophilia (blood eosinophils > 500 or ≤500 cells/µL), neutrophilia (blood neutrophils > 7700 or ≤7700 cells/µL), and CRP concentration (>5 or ≤5 mg/L). Giemsa-stained eosinophils were counted per square millimeter in the peritumoral and intratumoral regions of the lung cancer tissue as described in the Materials and Methods. Mean ± SEM values are shown. Significant differences between and within the groups were tested using the Mann–Whitney U-test. Only significant differences are shown: * p < 0.05; *** p < 0.001. The colored dots in (**C**,**D**) represent the number of patients and the representative tumor stage.

We observed a significantly higher infiltration of eosinophils into the peritumoral region of non-adeno-LC compared with adeno-LC (*p* < 0.05), regardless of eosinophilia or neutrophilia (Figure 4A). However, this effect was not observed in the intratumoral region (Figure 4B). 

Patients with adeno-LC and eosinophilia exhibited significantly higher eosinophil infiltration into the peritumoral region (*p* < 0.001) compared with those with neutrophilia or normal blood eosinophil and neutrophil counts (Figure 4C). Likewise, patients with non-adeno-LC with eosinophilia demonstrated significantly higher eosinophil infiltration into the peritumoral region compared with those with normal eosinophil and neutrophil counts (*p* < 0.05). However, no significant differences were observed compared with those with neutrophilia (Figure 4C). Additionally, patients with non-adeno-LC and neutrophilia showed a significant increase in eosinophil infiltration into the peritumoral region compared with those with adeno-LC and neutrophilia (*p* < 0.05; Figure 4C). These data suggest that non-adeno-LC is associated with increased infiltration of eosinophils into the peritumoral region. Moreover, it appears that blood eosinophilia may have an impact on local TME inflammatory response involving eosinophils in non-adeno-LC. Despite this, no substantial differences in the intratumoral infiltration of eosinophils were observed when comparing within or between different histological subgroups (Figure 4D). 

Infiltration of T cells into the intratumoral region was significantly induced in adeno-LC compared with non-adeno-LC, regardless of blood eosinophilia or neutrophilia (Figure S2A). Contrastingly, no difference was observed for T-cell infiltration between adeno-LC and non-adeno-LC within the peritumoral region (Figure S2B). The infiltration of mast cells was similar in the tumor tissue of patients with adeno-LC and non-adeno-LC, regardless of tumor location (peri vs. intratumoral) or peripheral blood eosinophilia/neutrophilia (Figure S2C,D). A microphotography of stained lung tumor tissue is provided in (Figure S3A,B).

## 3. Discussion

Lung cancer frequently occurs in patients with comorbidities that can complicate diagnoses and treatment [21]. In the context of chronic pulmonary inflammation, a link has been established between asthma/COPD comorbidities and elevated levels of circulating inflammatory mediators [22,23]. 

Our study reveals that the incidence of sIgE against food and inhalant allergens is significantly higher among patients with adeno-LC compared with non-adeno-LC. By contrast, individuals with non-adeno-LC exhibit higher levels of hs-CRP when compared with those with adeno-LC. Alterations in circulating inflammatory mediators, such as IgE or hs-CRP, manifest in shifts in peripheral inflammation dominated by eosinophil or neutrophil inflammation, respectively [19,24,25]. Our research supports the notion that peripheral inflammation featuring eosinophilia or neutrophilia is associated with survival outcomes in NSCLC, regardless of histological subgroup. However, a clear difference was observed between adeno-LC and non-adeno-LC histological subgroups, which is likely to influence the treatment plan. 

Blood eosinophilia resulted in the induction of eosinophil infiltration into the peritumoral region of the lung tissue, suggesting the presence of local, eosinophil-dominated inflammation that could alter the TME. Therefore, further research is necessary to thoroughly elucidate the link between blood eosinophilia and the localized inflammation that is driven by eosinophils within the tumor tissue. 

No direct connection has been established between the incidence of sensitization and increased risk of lung cancer. Several studies have shown that asthma history is associated with an increased risk of lung cancer, regardless of confounding variables, such as smoking [26,27,28,29]. Our initial cohort data reveal that individuals with adeno-LC had a higher occurrence of sIgE positivity than those with non-adeno-LC, indicating a greater prevalence of atopy in adeno-LC patients. It has been suggested that the activation of sIgE binding to its FcεRI receptor within tissues may yield superior anti-tumor responses compared with IgG engagement [30]. Allergic sensitization is primarily mediated through TH2 cells, which stimulate the production of sIgE antibodies and regulate the biology and function of eosinophils. TH2 cells, together with sIgE, play a crucial role in the maturation, recruitment, and survival of eosinophils, which are key effector cells in the type 2 immune response to allergens. Eosinophilia can be used as a reliable biomarker of type 2 immune inflammation [21,22,23]. Different hypotheses have been proposed that link IgE and Th2 cells to a tumor-promoting phenotype in cancer. IgE-mediated atopic reactions in the lung may generate a tumor-promoting environment. This environment includes the infiltration of inflammatory cells such as eosinophils and neutrophils, cytokine release, and genetic/epigenetic instability. Additionally, allergic reactions can shift the immune response from TH1-type (tumor-eradicating) to TH2-type responses. This shift suppresses the necessary cytolytic and inflammatory responses that enhance the elimination of lung tumor cells and promote a Treg phenotype in the lung [31]. Conversely, a reduction in the ability of immune cells to kill tumor cells can be attributed to allergens binding to the IgE receptors on these cells, effectively blocking the binding of tumor antigens. This competition for the IgE receptors hampers the immune response against the tumor [32]. 

It is well known that serum hs-CRP is induced in asthmatic and COPD individuals compared with healthy controls [19,24,25,26] as well as in patients with lung cancer [27]. The well-known inflammatory marker hs-CRP has been linked to both asthma [19,33,34] and lung cancer [35]. Numerous studies have demonstrated its role as a predictor of survival rates for cancer patients [15,36,37]. However, a study by Muller et al. found no association between circulating hs-CRP concentrations and lung adenocarcinoma [38]. We have shown that individuals with non-adeno-LC have significantly higher hs-CRP levels compared with those with adeno-LC, indicating underlying chronic inflammation. However, a group of patients with only COPD or asthma without lung cancer was not included, as our study is specific to lung cancer patients. Additionally, patients with asthma or COPD are typically treated in outpatient settings and are only hospitalized during exacerbations. Therefore, including this group would introduce confounding variables and lead to unmatched comparisons. These differences led us to assess the association between peripheral inflammation and survival of patients with NSCLC with distinct histological subtypes, primarily adeno-LC and non-adeno-LC. 

Eosinophils may exhibit both boosting and attenuating effects on the immune system, based on the type of cancer and different factors within the TME [38,39,40]. The non-beneficial impact of eosinophils has also been observed in cancer [41,42]. In the presence of airway allergy, eosinophilia promoted tumor cell migration and metastasis in mice [43]. Contrastingly, circulating neutrophils act as indicators of host inflammation, which is a main clinical feature observed in cancer patients [44]. A high proportion of circulating neutrophils compared with lymphocytes is considered a biomarker of poor clinical outcomes in several types of cancer [45]. Furthermore, the impact of eosinophilia and neutrophilia in malignancies is gaining more attention [46,47]. However, it remains unclear how eosinophilia and neutrophilia impact different histological subtypes of NSCLC. 

In general, our findings indicate that both eosinophilia and neutrophilia in patients with NSCLC might be associated with a lower survival rate when compared with normal eosinophils or neutrophils in blood, respectively. Our study reveals that blood eosinophilia is associated with poorer survival preferentially in the advanced stages (III and IV) of patients with adeno-LC, but not in patients with non-adeno-LC. Moreover, blood neutrophilia is associated with poorer survival of patients in advanced NSCLC stages (III and IV) in both histological subgroups. 

Multivariate analyses demonstrated that eosinophilia, neutrophilia, or a combination of both might predict the outcome of patients with adeno-LC. However, neutrophilia alone can predict the outcome of non-adeno-LC. These findings might provide new insight into the optimal treatment strategy for NSCLC, considering both peripheral inflammation levels (eosinophilia and neutrophilia) and the histological subtype of NSCLC (adeno-LC and non-adeno-LC). Additionally, it has been reported that circulating neutrophils are associated with tumor progression and treatment efficacy in patients with NSCLC [48,49]. 

Together, these results highlight the potential role of blood eosinophil and neutrophil counts as prognostic factors in both adeno-LC and non-adeno-LC. Given that neutrophilia can occur in patients with pneumonia, a significant limitation of our lung cancer cohort is the absence of data on the presence or absence of pneumonia at the time of diagnosis. The lung cancer and histology cohort excluded data from healthy volunteers, as they do not visit lung cancer clinics. This study specifically aims to examine peripheral inflammatory cells in lung cancer patients with various histological backgrounds. Additionally, obtaining lung tissue from healthy controls for studying immune cell infiltration is not ethically acceptable.

Compelling evidence suggests that the progression and outcomes of lung cancer might be associated with the distribution and the activity of the tumor-infiltrated immune cells within the stromal compartment of the tumor [50]. Our data show that T-cell and mast-cell distributions in the tumor seem to remain unchanged, regardless of the tumor’s histological subtype or location. By contrast, eosinophil infiltration into the peritumor region was significantly higher in adeno-LC with blood eosinophilia when compared with normal blood counts or neutrophilia. This pattern was also observed in non-adeno-LC when comparing the eosinophilia group with those with normal blood counts. The infiltration of immune cells into peritumoral and intratumoral areas plays a crucial role in determining cancer progression and patient survival. The interaction between stromal cells in the peritumoral area and immune cells in the intratumoral can influence tumor behavior and invasiveness. For example, in breast cancer, peritumoral immune cell infiltration is associated with cancer progression [51], whereas in pancreatic ductal adenocarcinoma (PDAC), intratumoral infiltration of immune cells such as CD3, CD4, and CD8 T cells is significantly correlated with better prognosis [52]. Therefore, studying the infiltration of immune cells in both peritumoral and intratumoral areas can contribute to more accurate predictions of cancer prognosis. Our study suggests the presence of localized inflammation in the peritumoral region, which is likely driven by eosinophil infiltration, in both adeno-LC and non-adeno-LC with blood eosinophilia. Further analysis using human and mouse models of lung cancer is required to understand the underlying mechanisms by which infiltrated eosinophils orchestrate the tumor microenvironment. Eosinophils may play a significant role in modulating immune responses and influencing the behavior of other cells within the tumor microenvironment.

## 4. Methods

### 4.1. Study Design and Study Population

Our retrospective study used the Data Warehouse of the German Center for Lung Research, the Hospital Information System (HIS), the clinical tumor registry at Thoraxklinik Heidelberg, and the Lung Biobank Heidelberg database as primary data sources to identify suitable patients and samples. Formalin-fixed paraffin-embedded samples from patients with NSCLC were resected at the Thoraxklinik in Heidelberg and provided by the National Center for Tumor Diseases Tissue Bank. All patients gave written informed broad consent for the use of their samples for scientific purposes (ethical votes S-270/2001 and S-071/2020, Ethics Committee Heidelberg).

Three cohorts were analyzed:

1. Lung cancer cohort: Retrospective, including 3,143 patients with NSCLC diagnosed between 2010 and 2020. Of these, 2,113 had adeno-LC and 1030 had non-adeno-LC, which was primarily small squamous cell carcinoma histology. Inclusion criteria: (1) histological classification (adeno-LC and non-adeno-LC) according to the WHO Classification of Lung Tumors [53]; and (2) available data on pre-therapeutic differential white blood cell count. This cohort was used to study the association of blood eosinophilia and neutrophilia and survival of patients diagnosed with NSCLC (adeno-LC or non-adeno-LC). The demographic data are detailed in Table 1.
2. Asthma/COPD comorbidity sub-cohort: A subgroup of 212 NSCLC patients was selected from the Lung Biobank Heidelberg cancer cohort, of which 115 had adeno-LC and 97 had non-adeno-LC. Inclusion criteria: (1) asthma (n = 48) or COPD (n = 90) comorbidities; and (2) available pre-therapeutic serum samples. A control group (n = 74) comprised patients with NSCLC without a diagnosis of asthma or COPD. The three groups (no asthma or COPD; COPD; asthma) were matched for gender, age, histology, stage, and date of diagnosis; the demographic data are detailed in Table 4. This sub-cohort was used to study sensitization and subclinical inflammation in adeno-LC and non-adeno-LC, by measuring the sIgE and hs-CRP.
3. Tissue eosinophilia sub-cohort: Selected to assess the infiltration of immune cells (eosinophils, mast cells, and T cells) into the peritumoral and intratumoral regions. Inclusion criteria: (1) available formalin-fixed paraffin-embedded tissue sample from resected patients; and (2) pre-surgical white blood cell count. Six groups were analyzed (n ≥ 9 patients each) based on histology (adeno-LC vs. non-adeno-LC), blood eosinophil count (split at 500 cells/µL), neutrophil count (split at 7700 cells/µL), and blood CRP level (split at 5 mg/l). Details on the groups’ distribution are provided in Table 5.

### 4.2. Blood Measurements from the Asthma/COPD Comorbidity Cohort 

Blood samples (N = 212) were analyzed at the *Institute* of *Laboratory Medicine* and Pathobiochemistry, University Hospital Marburg, for inflammatory markers, including hs-CRP and a range of specific IgE antibodies against representative panel of food and inhalant allergens. IgE antibodies in patient serum were measured using ImmunoCAP™ (Thermo Fisher Scientific, Waltham, MA, USA). The ImmunoCAP™ platform is a sandwich assay, which reports kilounits per liter (kU/L) (calibrated against the WHO standard for IgE). Two premixed assays were used including specific IgE antibodies against inhalant allergens and food allergens. The sx1 mix contained eight inhalant allergen extracts (*Artemisia vulgaris*, *Betula verrucosa*, *Cladosporium herbarum*, *Dermatophagoides pteronyssinus*, dog dander, cat epithelium, *Phleum pratense*, cultivated rye). The fx5 mix comprised six food allergen extracts (cod, egg, peanut, cow’s milk, soya, wheat flour). A sample was considered positive if the patient had sIgE ≥ 0.35 kU/L against one or more of the allergens [54]. 

Using the clinical data and the measurements of eosinophil count and IgE (total and specific), patient groups were allocated further. Patients with comorbid type 2 inflammatory asthma were defined based on three criteria, namely, blood eosinophil count above 300 cells/µL, total IgE above 200 kU/L, and specific IgE above 0.35 kU/L. 

### 4.3. Immunohistochemistry and Giemsa Staining from the Tissue Eosinophilia Sub-Cohort

For immunohistochemistry, heat-induced epitope retrieval was carried out using citrate buffer [55]. Sections were stained on a Dako Autostainer Plus after blocking endogenous peroxidase activity and underwent a 45 min incubation period with mouse anti-human CD3 monoclonal antibody (mAb; clone F7.2.38, diluted 1:50) or rabbit anti-human CD117 polyclonal antibody (pAb; diluted 1:200) to stain T cells and mast cells, respectively. The sections were washed and incubated with Dako REAL EnVision HRP Rabbit/Mouse-labeled polymer according to the manufacturer’s protocol. Applying standard techniques, sections were also stained with Giemsa solution, using 1% acetic acid for differentiation. All antibodies were purchased from Agilent, Santa Clara, United States. 

A blinded evaluation of digitized histology and staining results was conducted. A total of 10 high-power fields (HPFs) were defined from tissue areas that contained the highest cell counts. Of these HPFs, five were located in the peritumoral region and five in the intratumoral region, excluding blood vessels and necrotic areas. QuPath bioimaging analysis software (version 0.3.2) was used to analyze T cells and mast cells, while Giemsa-stained eosinophils were counted manually.

### 4.4. Statistics

Statistical analyses of the lung cancer cohort and the asthma/COPD comorbidity sub-cohort were performed using SPSS 27. The Mann–Whitney U-test was used to compare two groups and the Kruskal–Wallis test was used for comparisons between multiple groups. Differences in distribution of parameters between groups were analyzed with the chi-squared test. Survival curves were calculated using the Kaplan–Meier method and compared with the log-rank test. Multivariate survival analyses were carried out with Cox regression analyses. A *p*-value of less than 0.05 was considered statistically significant. For the tissue inflammation sub-cohort, graphs were produced using Graph Pad Prism (version 9.4.1). 

The predictive value of eosinophilic and neutrophilic inflammation on the survival of new patients was evaluated by time-dependent Brier scores and time-dependent ROC analyses using bootstrap cross-validation with 100 bootstrap datasets. The calculations were carried out using R (version 4.2.1) [56] with the Risk Regression package (version 2022.11.28) [57].

## 5. Conclusions

Given our current findings, more attention should be paid to circulating eosinophils and neutrophils in patients with NSCLC, taking into account the different histological subgroups. Moreover, future clinical studies and animal models should be conducted to understand the role of infiltrated eosinophils in shaping the TME and treatment responses in NSCLC. 

## Figures and Tables

**Table 1:** Demographic data of the Lung Cancer Cohort.

Lung Cancer Cohort		Total	Non-Adeno-LC N (%)	Adeno-LC n (%)	*p*-Value
**Total**		3143 (100%)	1030 (100%)	2113 (100%)	N/A
**Sex**	**m**	1910 (60.8%)	759 (73.7%)	1151 (54.5%)	*p* < 0.001 *
	**f**	1233 (39.2%)	271 (26.3%)	962 (45.5%)	
**Age**	**Median (min, max)**	N/A	66 (40, 91)	65 (22, 90)	*p* = 0.001 **
**BMI**	**Median**		26 (100%)	25.4	*p* = 0.004 **
	**(min, max)**		(13.8, 54.1)	(14.7, 57.8)	
**Best Stage (if available *p*-Stage, otherwise c-Stage)**	**I**	522 (16.6%)	159 (15.4%)	363 (17.2%)	*p* < 0.001 *
	**II**	341 (10.8%)	174 (16.9%)	167 (7.9%)	
	**III**	885 (28.2%)	435 (42.2%)	450 (21.3%)	
	**IV**	1395 (44.4%)	262 (25.4%)	1133 (53.6%)	
**Smoking status**	**No**	336 (10.7%)	40 (3.9%)	296 (14%)	*p* < 0.001 *
	**Yes**	1105 (35.2%)	390 (37.9%)	715 (33.8%)	
	**Ex/former**	1534 (48.8%)	561 (54.5%)	973 (46%)	
	**N/A**	168 (5.3%)	39 (3.8%)	129 (6.1%)	
**Eosinophilia/Neutrophilia**	**Neutrophilia** (Neutrophils > 7700/µL and eosinophils ≤ 500/µL)	770 (24.5%)	289 (28.1%)	481 (22.8%)	*p* = 0.002 *
	**Eosinophilia** (Eosinophils > 500/µL and Neutrophils ≤ 7700/µL)	108 (3.4%)	41 (3.9%)	67 (3.2%)	
	**Eosinophilia and Neutrophilia** (Eosinophils > 500/µL and Neutrophils > 7700/µL)	75 (2.4%)	17 (1.7%)	58 (2.7%)	
	**No Eosinophilia or Neutrophilia** (Eosinophils ≤ 500/µL and Neutrophils ≤ 7700/µL)	2190 (69.7%)	683 (66.3%)	1507 (71.3%)	

3143 patients with non-small cell lung cancer (NSCLC) at Thoraxklinik Heidelberg diagnosed between 2010 and 2020 were included. Inclusion criteria were: (1) histology classification, adeno-LC (n = 2113) and non-adeno-LC (n = 1030); and (2) the availability of pre-therapeutic differential blood cell count data. Differences between histological groups were tested with * Chi2 test (qualitative variables) or ** median test (continuous variables), respectively. A *p*-value less than 0.05 was considered significant. N/A: not applicable; m: male; f: female; adeno: adenocarcinoma; non-adeno: non-adenocarcinoma; LC: Lung Cancer (NSCLC); n: number; (%): percentage.

**Table 2:** Result of multivariate survival analyses of NSCLC from the Lung cancer cohort.

		Adeno-LC			Non-Adeno-LC		
		Hazard Ratio	95%CI	*p* Multivariate	Hazard Ratio	95%CI	*p* Multivariate
**Age**		1.01	1.01–1.02	***1.43 × 10^−5^***	1.03	1.02–1.04	***1.29 × 10^−8^***
**Sex**		0.74	0.66–0.83	***4.23 × 10^−7^***	0.94	0.78–1.12	0.50
**Stage**	**I**	1			1		
	**II**	1.27	0.86–1.89	0.24	1.02	0.71–1.46	0.92
	**III**	3.48	2.64–4.58	***7.68 × 10^−19^***	2.69	2.01–3.58	***1.90 × 10^−11^***
	**IV**	7.70	5.95–9.95	***1.07 × 10^−54^***	6.27	4.64–8.47	***5.92 × 10^−33^***
**BMI**		1.00	0.99–1.01	0.87	0.98	0.96–0.99	***0.0099***
**Smoking status**	**N0**	1			1		
	**Ex/former**	1.35	1.16–1.58	***0.0001***	0.87	0.66–1.14	0.31
	**Yes**	1.50	1.28–1.77	***1.05 × 10^−6^***	1.03	0.78–1.36	0.86
**EOS/Neutros**	**No Eosinophilia or Neutrophilia**	1			1		
	**Neutrophilia**	1.39	1.22–1.57	***5.04 × 10^−7^***	1.55	1.30–1.84	***6.64 × 10^−7^***
	**Eosinophilia**	1.35	1.01–1.81	***0.044***	0.95	0.64–1.41	0.80
	**Eosinophilia and Neutrophilia**	2.03	1.51–2.72	***2.45 × 10^−6^***	1.34	0.71–2.52	0.37

Cox regression analyses (inclusion model) controlling for age, gender, stage, BMI, smoking status, and peripheral blood eosinophil and/or neutrophil count. adeno: adenocarcinoma; non-adeno: non-adenocarcinoma; LC: Lung Cancer (NSCLC); *p*-value was calculated as described in the Section 4, bold and italics used for significant *p*-values.

**Table 3:** Result of multivariate survival analyses of NSCLC from the Lung cancer cohort with respect to peripheral inflammation.

	All NSCLC			
	**n**	**MS (mos)**	**5-yr-S (%)**	***p*** **(Multivariate)**
**Normal Eosinophils and Neutrophils**	2190	28.0	29.0	
**Neutrophilia**	770	13.4	19.0	3.0506 × 10^−23^
**Eosinophilia**	108	16.8	16.5	0.003
**Eosinophilia and Neutrophilia**	75	8.2	n.def.	1.4714 × 10^−9^
	**Adeno-LC**			
	**n**	**MS (mos)**	**5-yr-S (%)**	***p*** **(Multivariate)**
**Normal Eosinophils and Neutrophils**	1507	29.5	29.2	
**Neutrophilia**	481	13.4	19.5	5.04 × 10^−7^
**Eosinophilia**	67	16.1	n.def.	0.044
**Eosinophilia and Neutrophilia**	58	5.7	n.def.	2.45 × 10^−6^
	**Non-Adeno-LC**			
	**n**	**MS (mos)**	**5-yr-S (%)**	***p*** **(Multivariate)**
**Normal Eosinophils and Neutrophils**	683	25.5	28.6	
**Neutrophilia**	289	13.5	17.8	6.64 × 10^−7^
**Eosinophilia**	41	18.0	24.7	0.80
**Eosinophilia and Neutrophilia**	17	14.6	n.def.	0.37

Results of multivariate analyses with respect to count of peripheral eosinophils and neutrophils. Overview on median survival (MS) in months (mos) and 5-year survival rate in % (5-years). n.def.: not defined.

**Table 4:** Demographic data of the Asthma/COPD Comorbidity Cohort.

		Total	NSCLC No Asthma/No COPD N (%)	NSCLC COPD N (%)	NSCLC Asthma N (%)
**Patients number**		212 (100%)	74 (34.9%)	90 (42.5%)	48 (22.6%)
**Histology** **(% from Histology)**	**adeno-LC**	115 (54.2%)	42 (56.8%)	38 (42.2%)	35 (72.9%)
	**non-adeno-LC**	97 (45.8%)	32 (43.2%)	52 (57.8%)	13 (27.1%)
**Sex**	**m/f**	101/111	36/38	47/43	18/30
**Age**	**Median** **(min-max)**	63 (39–87)	64 (44–87)	63 (47–80)	62.5 (39–84)
**BMI**	**Median**	25.8	25.8	25.6	26.2
**Stage (% from comorbidity group)**	**1/2**	128 (60.4%)	34 (45.9%)	64 (71.1%)	30 (62.5%)
	**3/4**	84 (39.6%)	40 (54.1%)	26 (28.9%)	18 (37.5%)
**Smoking in patient history**	**yes (ever)**	183	61	87	35
	**no**	21	11	1	9
	**n/a**	8	2	2	4

212 NSCLC patients were included. The inclusion criteria were based on: (1) the presence of lung comorbidity diagnosis including asthma alone (n = 48) or COPD alone (n = 90); (2) and the presence of bio samples in the Lung Biobank Heidelberg (blood/tissue). The control group contains NSCLC patients (n = 74) without a history of either asthma or COPD. NSCLC patients were classified according to histology adeno-LC (n = 115) and non-adeno (n = 97) and the presence of lung comorbidities (asthma alone, COPD alone, no asthma/no COPD). Patient groups were matched by gender, age, stage, cancer diagnosis, and primary tumor histology. m: male; f: female; adeno: Adenocarcinoma; non-adeno: non-adenocarcinoma; NSCLC: non-small-cell lung cancer; N: number; (%): percentage.

**Table 5:** Distribution of NSCLC tumor tissue samples, according to the histology type (adeno- and non-adeno-LC) and with respect to the blood eosinophil, neutrophil counts, and the blood CRP concentration.

	Blood			
NSCLC	N	Eosinophils (cells/µL)	Neutrophils (cells/µL)	CRP (mg/L)
**adeno-LC**	10	>500	≤7700	≤5
**non-adeno-LC**	10	>500	≤7700	≤5
**adeno-LC**	9	≤500	>7700	>5
**non-adeno-LC**	10	≤500	>7700	>5
**Control adeno-LC**	10	≤500	≤7700	≤5
**Control non-adeno-LC**	10	≤500	≤7700	≤5

## Data Availability

Data of the Asthma/COPD comorbidity cohort will be transferred to the data warehouse of the German Center for Lung Research (DZL) for potential further scientific use. An access to the data derived from the clinical lung cancer registry of the Thoraxklinik cannot be granted to external persons due to data protection and ethical issues. Registry data are not allowed to leave the clinic for external storage.

## Supplementary Materials

The following supporting information can be downloaded at: https://www.mdpi.com/article/10.3390/ijms25179552/s1.

## Abbreviations

NSCLC	non-small cell lung cancer
TH2	T helper type 2
hs-CRP	high-sensitivity C-reactive protein
adeno-LC	adenocarcinoma lung cancer
non-adeno-LC	non- adenocarcinoma lung cancer
ZfKD	German *Centre for Cancer Registry Data*
COPD	chronic obstructive pulmonary disease
sIgE	allergen-specific immunoglobulin E
HIS	Hospital Information System
HPFs	high-power fields
BMI	body mass index
HR	Hazard Ratio
TME	tumor microenvironment
PDAC	pancreatic ductal adenocarcinoma
