# Supplementary material for: Peripheral Inflammation Featuring Eosinophilia or Neutrophilia Is Associated with the Survival and Infiltration of Eosinophils within the Tumor among Various Histological Subgroups of Patients with NSCLC
Source: Int J Mol Sci. 2024 Sep 3;25(17):9552. doi: 10.3390/ijms25179552 (PMC11395097; doi:10.3390/ijms25179552)
Supplement: Supplementary file 1 [file ijms-25-09552-s001.zip › ijms-3077847-supplementary.pdf]

Peripheral Inflammation Featuring Eosinophilia or Neutrophilia Is Associated with the Survival and Infiltration of Eosinophils within the Tumor among Various Histological Subgroups of Patients with NSCLC

Bilal Alashkar Alhamwe [1,2,3,*,†], Kadriya Yuskaeva [4,5,‡], Friederike Wulf [1], Frederik Trinkmann [4,6,7], Mark Kriegsmann [4,8], Michael Thomas [4,9], Corinna Ulrike Keber [10], Elke Pogge von Strandmann [2], Felix J. Herth [4,6], Saeed Kolahian [1], Harald Renz [1,‡] and Thomas Muley [4,5,*,†]

[1] Institute of Laboratory Medicine, German Center for Lung Research (DZL), Universities of Giessen and Marburg Lung Center (UGMLC), Philipps University of Marburg—Medical Faculty, 35043 Marburg, Germany; friederike.wulf@e-mail.de (F.W.); saeed.kolahian@uni-marburg.de (S.K.); harald.renz@uk-gm.de (H.R.)
[2] Institute of Tumor Immunology, Center for Tumor Biology and Immunology, Philipps University Marburg, 35043 Marburg, Germany; poggevon@staff.uni-marburg.de, bilal.alashkaralhamwe@staff.uni-marburg.de
[3] College of Pharmacy, International University for Science and Technology (IUST), Daraa 15, Syria
[4] Translational Lung Research Center (TLRC), German Center for Lung Research (DZL), 35394 Heidelberg, Germany; kadriya.yuskaeva@med.uni-heidelberg.de (K.Y.); frederik.trinkmann@med.uni-heidelberg.de (F.T.); kriegsmann@pathologie-wiesbaden.de (M.K.); michael.thomas@med.uni-heidelberg.de (M.T.); felix.herth@med.uni-heidelberg.de (F.J.H.)
[5] Translational Research Unit (STF), Thoraxklinik, University Hospital Heidelberg, 69126 Heidelberg, Germany
[6] Department of Pneumology and Respiratory Medicine, Thoraxklinik, University Hospital Heidelberg, 69126 Heidelberg, Germany
[7] Department of Biomedical Informatics (DBMI), Center for Preventive Medicine and Digital Health Baden-Württemberg (CPD-BW), University Medical Center Mannheim, Medical Faculty Mannheim, Heidelberg University, 69117 Heidelberg, Germany
[8] Institute of Pathology, University Hospital Heidelberg, Pathology Wiesbaden, Ludwig-Erhard-Str. 100, 65199 Wiesbaden, Germany
[9] Department of Oncology, Thoraxklinik, University Hospital Heidelberg, 69126 Heidelberg, Germany
[10] Institute for Pathology, University Hospital Giessen and Marburg, 35037 Marburg, Germany; brehmc@med.uni-marburg.de

* Correspondence: bilal.alashkaralhamwe@staff.uni-marburg.de (B.A.A.); thomas.muley@med.uni-heidelberg.de (T.M.); Tel.: +49-6421-28-21641 (B.A.A.); +49-6221-396-1110 (T.M.)

† These authors contributed equally to this work and share the corresponding.
‡ These authors contributed equally to this work.

Citation: Alashkar Alhamwe, B.; Yuskaeva, K.; Wulf, F.; Trinkmann, F.; Kriegsmann, M.; Thomas, M.; Keber, C.U.; Strandmann, E.P.v.; Herth, F.J.; Kolahian, S.; et al. Peripheral Inflammation Featuring Eosinophilia or Neutrophilia Is Associated with the Survival and Infiltration of Eosinophils within the Tumor among Various Histological Subgroups of Patients with NSCLC. *Int. J. Mol. Sci.* **2024**, *25*, x. https://doi.org/10.3390/xxxxx

Academic Editor(s): Name

Received: 11 June 2024
Revised: 20 August 2024
Accepted: 26 August 2024
Published: date

Copyright: © 2024 by the authors. Submitted for possible open access publication under the terms and conditions of the Creative Commons Attribution (CC BY) license (https://creativecommons.org/licenses/by/4.0/).

Supplementary Figures

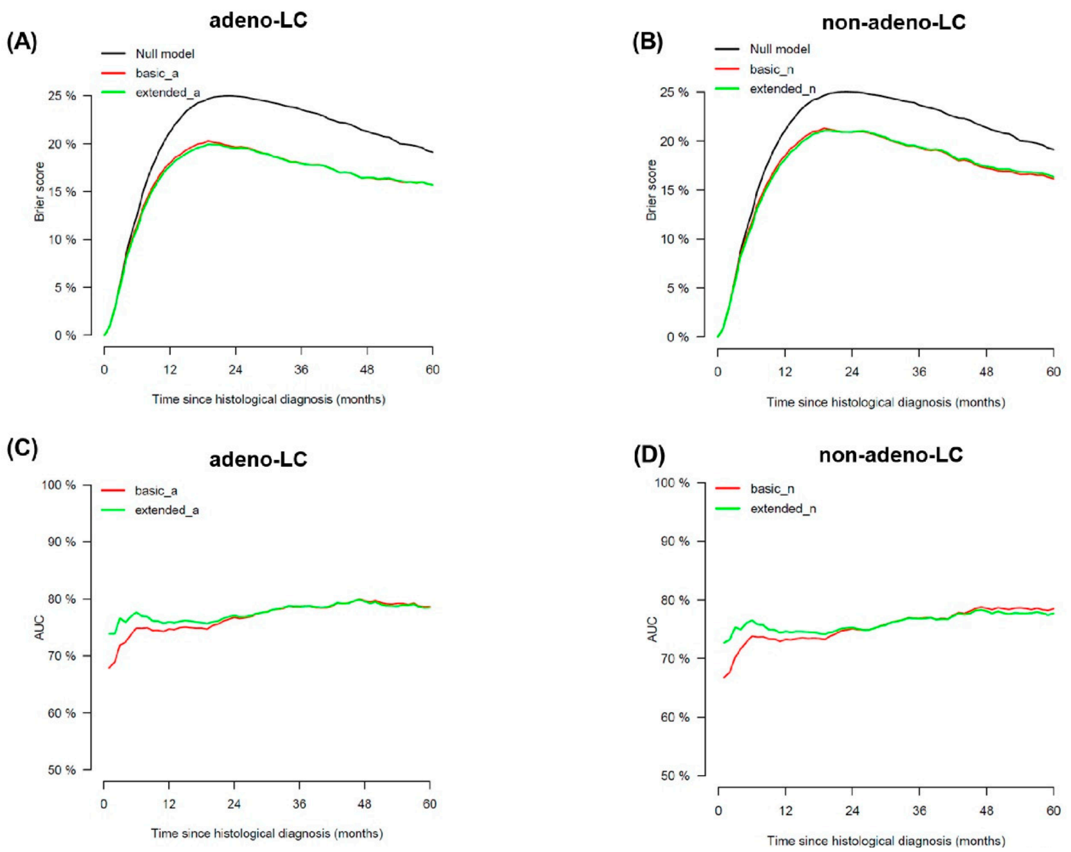

Supplementary Figure S1. Predictive ability of eosinophilia/neutrophilia on survival in NSCLC histological subgroups. (A–D) The predictive ability of eosinophil/neutrophil data on survival was calculated by time-dependent prediction error (Brier score; A,B) and time-dependent AUC curves (C,D). In A and B the null model describes the prediction without information. (A) Basic_a includes the clinical information of patients with adeno-LC, extended_a includes the clinical information plus the eosinophil/neutrophil cut-off counts. (B) Basic_n includes the clinical information of patients with non-adeno-LC, extended_n includes the clinical information plus eosinophil/neutrophil cut-off counts. In C and D the predictive ability of eosinophilia/neutrophilia is calculated with respect to the time-dependent AUC analyses. In C, basic_a includes the clinical information of patients with adeno-LC,

and extended_a includes the clinical information plus the eosinophil/neutrophil cut-off counts. In D, basic_n includes the clinical information of patients with non-adeno-LC, and extended_n includes the clinical information plus the eosinophil/neutrophil cut-off counts.

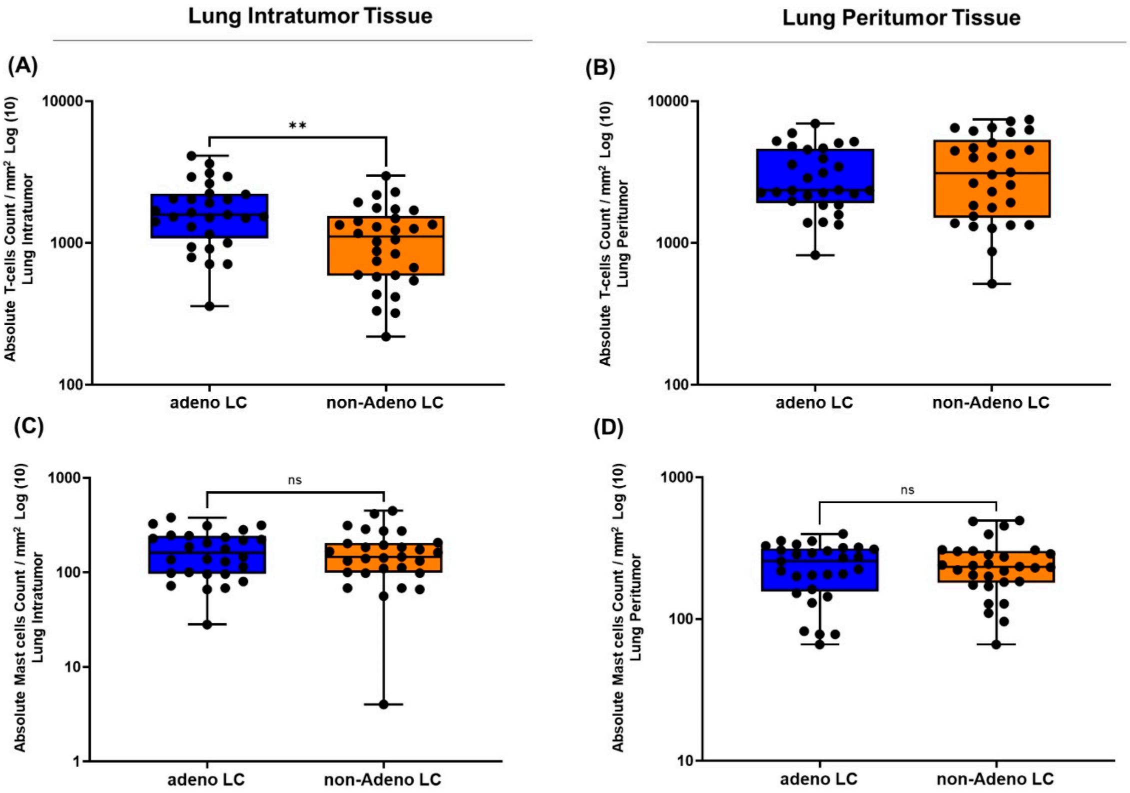

Supplementary Figure S2. Distribution of T cells and mast cells in lung cancer tissue. The distribution of infiltrated T-cells and mast cells was tested in respect to histology (adeno-LC vs. non-adeno-LC), regardless of the eosinophilia or neutrophilia status in blood. Anti-CD117 (mast cells) and anti-CD3 (T cells) positive cells were counted per square millimeter in the peritumoral and intratumoral regions of the LC tissue as described in the Materials and Methods. (A,B) The absolute number of T cells in the intratumoral (A) and peritumoral (B) regions. (C,D) The absolute number of mast cells in the intratumoral (C) and peritumoral (D) regions. Mean ± SEM values are shown. Significant

differences between the groups were tested using the Mann–Whitney U-test. Each dot represents apatient. **$p < 0.01$.

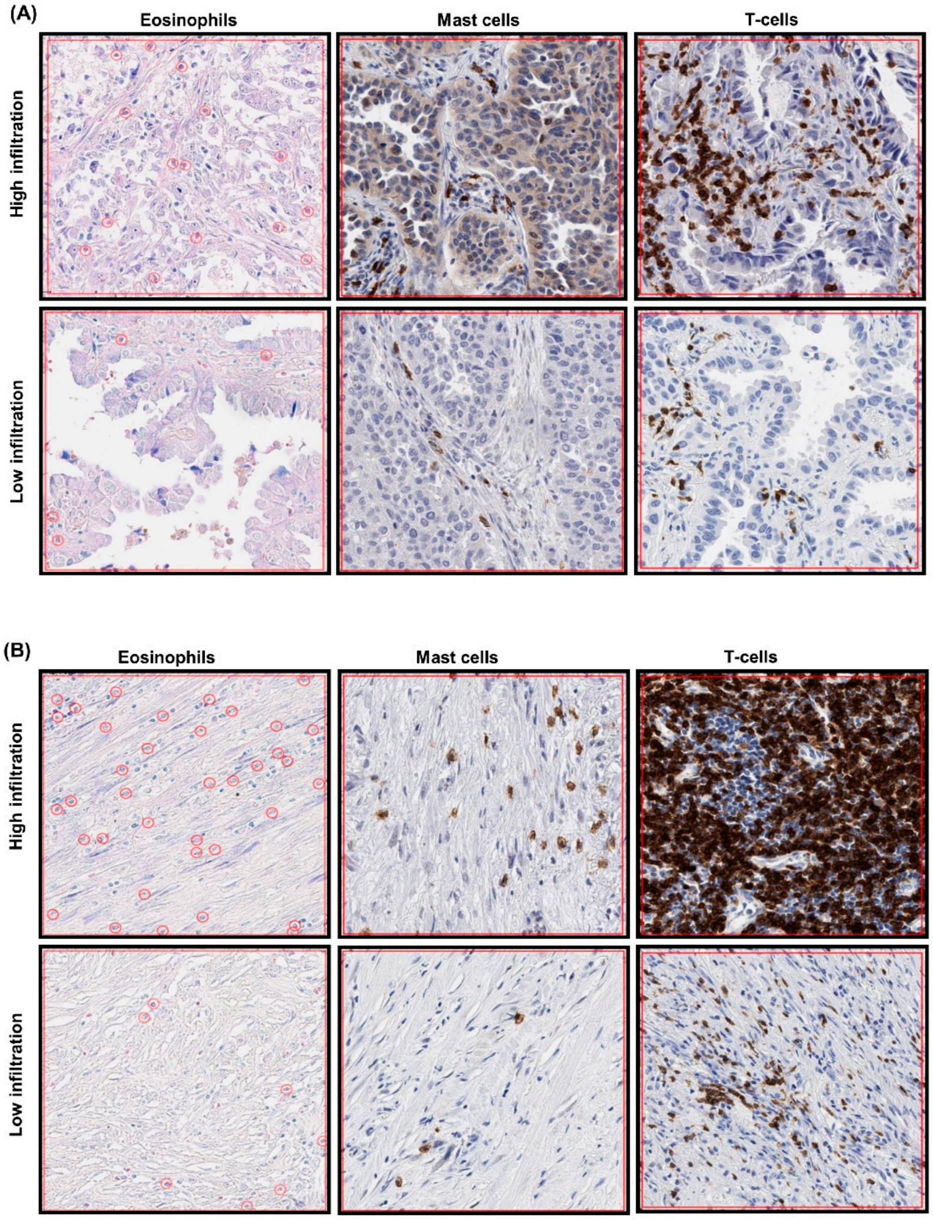

Supplementary Figure S3. Representative pictures of one HPF with high infiltrated or low infiltrated immune cells. (A) Representative peritumoral pictures of one HPF in respect to high or low infiltrated cells. (B) Representative intratumoral pictures of one HPF in respect to high or low

infiltrated cells. Cells in A and B were stained with Giemsa for eosinophils, anti-CD117 for mast cells, and anti-CD3 for T cells
